# Clear Aligner Treatment in Denmark – A Questionnaire Survey among Citizens and Dental Practitioners in Denmark

**DOI:** 10.2340/aos.v85.45996

**Published:** 2026-05-11

**Authors:** Clara Rasborg Hartogsohn, Kasper Rosing, Liselotte Sonnesen

**Affiliations:** a Orthodontics, Section for Oral Health, Society and Technology, Department of Odontology, Faculty of Health and Medical Sciences, University of Copenhagen, Copenhagen, Denmark; b Community Dentistry, Section for Oral Health, Society and Technology, Department of Odontology, Faculty of Health and Medical Sciences, University of Copenhagen, Copenhagen, Denmark

**Keywords:** Orthodontics, clear aligner treatment, malocclusion

## Abstract

**Objectives:**

This study investigates clear aligner treatment (CAT) of adult patients in private dental practice and aims to identify: 1) The awareness of and knowledge about CAT among citizens, 2) how citizens who have received CAT experience the treatment and 3) how general dentists and orthodontists assess CAT.

**Materials and methods:**

The study was based on an observational cross-sectional questionnaire investigation. Two separate online questionnaires were sent out to all private dental clinics in Denmark and to a group of 2000 citizens aged ≥18 years.

**Results:**

217 citizens and 200 practitioners were included in the analysis. The citizens’ awareness of CAT was poor in general, and younger citizens were more aware of CAT. The citizens most often became aware of CAT through their own dentist or through friends and family. The citizens were in general satisfied with their treatments.The practitioners always/often informed about all parameters possible. The most frequent indication for CAT was cosmetics. The orthodontists more often included a lateral cephalogram in the diagnostics before CAT and more often made changes and more comprehensive changes to the digital treatment plan. The orthodontists were less satisfied with CAT compared to the general dentists.

**Conclusions:**

This study has provided valuable knowledge on the awareness and satisfaction of CAT among the citizens and indicated how practitioners assess CAT of adult patients in private dental practice in Denmark.

## Introduction

Since the introduction of Invisalign® (Santa Clara, California) in 1997, the interest in and demand for clear aligner treatment (CAT) has been increasing [1]. In Denmark, orthodontic treatment is performed by both orthodontists and by general dentists. However, it is stated in the ‘Legal Notice on Dental Care’ [2] that orthodontic treatment should aim to be carried out by specialists in orthodontics, and in case different dental care groups are involved in the treatment, it should clearly be stated in the patient’s record how the responsibilities are distributed. Lately, CAT is growing in popularity among the general dentists concurrently with an increase in demand from the patients and marketing from the distributers [3].

Several studies indicate that patients are choosing CAT over fixed appliance treatment (FAT) mainly due to aesthetic reasons [4–11]. Aside from the aesthetics, patients also tend to experience fewer inconveniences, a better oral health-related quality of life (OHRQoL) and in general experience a more pleasant treatment when receiving CAT compared to FAT [4, 6, 12–14]. However, there are also studies [12, 15] indicating minimal or no difference at all regarding these aspects between the two types of appliances. To this date, only a few studies have investigated the patients’ experience with CAT in relation to the course of treatment as well as the results of the treatment [6, 16, 17]. To our knowledge, no studies have investigated the Danish citizens’ awareness of CAT and how the citizens experience the treatment as well as how the practitioners in Denmark assess CAT.

As for all other orthodontic treatments, correct diagnostics and well-considered treatment planning are essential and the decisions that are made should be based on solid knowledge about biological and biomechanical principles [18]. Prior to CAT, the practitioner ought to perform a thorough anamnesis, a clinical examination, collect relevant radiographic material as well as other kinds of material needed for diagnostics and treatment planning [18].

Prior to CAT, the patient must consent to the treatment on a well-informed basis [19]. The practitioner is responsible for making sure that the patient receives proper information prior to treatment such as possible side effects, complications related to the treatment and alternative treatment options.

Based on the existing literature [1, 16, 20], the current indications for CAT are:

- Mild to moderate dentoalveolar malocclusions where there is no need for extractions, extrusions, major rotations or root movements.
- Minor relapses after previous orthodontic treatment.

According to the World Health Organization (WHO), health is defined as “(…) a state of complete physical, mental and social well-being and not merely the absence of disease or infirmity” [21]. In general, there is no agreement what health needs are built on and a need for a certain treatment can be perceived differently among practitioners and patients or even among practitioners alone [22]. Needs can be influenced by contemporary culture in society while the demand for a certain type of treatment might be influenced by social factors and the educational background of an individual as well as influence from media or health care professionals [23].

Given the growing interest in CAT, it was deemed interesting to investigate the current status in the treatment of adult patients in private dental care among citizens and practitioners. The aims of the study were to investigate:

- The occurrence of citizens who are aware of CAT or who have received CAT
- Where the citizens get their knowledge about CAT and what their opinion on CAT is influenced by
- How citizens who have received CAT experience the treatment
- How general dentists and orthodontists assess CAT

The hypotheses of the present study were:

- The need for CAT among the citizens has been increasing and is most often based on cosmetics
- The citizens most often get their knowledge and opinions on CAT from other sources than health professionals
- Not all general dentists have sufficient professional knowledge and competences about CAT

## Materials and methods

This project was registered with the Danish Data Protection Agency (514-0887/23-3000) prior to startup. The participants have agreed to participate in the study. No approval from the ethics committee was needed due to the study type.

This observational and cross-sectional study used questionnaire surveys as a method for data collection. The study consisted of two separate online questionnaires to the citizens and the practitioners, respectively. The questionnaires were constructed using SurveyXact (Rambøll, Copenhagen) and were under construction from August 2023. The questionnaires were continuously adjusted and improved until distribution in October 2023. The online questionnaires were open for responses from October 2023 to February 2024.

### In- and exclusion Criteria

The following in- and exclusion criteria were listed for the citizens:

Inclusion:

- Adult (≥18 years of age) citizens with a Danish civil registration number
- Completed responses

Exclusion:

- Partial completed responses

The following in- and exclusion criteria were listed for the practitioners:

Inclusion:

- General dentists working in private dental practice (adult patients)
- Orthodontists working in private dental practice (adult patients)
- Completed responses

Exclusion:

- General dentists working in municipal and regional dental care
- Orthodontists working in municipal and regional dental care
- Partial completed responses

### Pilot Testing

Both questionnaires underwent pilot testing prior to distribution. The citizen questionnaire was tested by three different non-professional individuals, two females and one male of different ages (25-64 years old), location and occupation to test whether the questions were clear and easy to understand and to test whether the questionnaire was easy to use. The practitioner questionnaire was tested by three dental professionals, two females and one male, aged 46-51 years old: an orthodontist, a general dentist who provided CAT and a general dentist, who did not provide CAT. Both questionnaires were tested by people with Danish as their native language because the questionnaires were available only in Danish.

### Questionnaires

There were three different paths in the citizens’ questionnaire:

- Citizens who have received/are receiving CAT
- Citizens who are uncertain about having a need for CAT
- Citizens who are certain about not having a need for CAT

In the questionnaire for the practitioners, there were two different paths:

- Practitioners who provide CAT
- Practitioners who do not provide CAT

For those providing CAT, the questions were regarding different aspects of the treatment such as indications and demand, information prior to treatment, diagnostic material, treatment planning, educational background, satisfaction with treatment results, etc. in order to clarify how the practitioners assess CAT. The practitioners who do not provide CAT were asked about why they had chosen not to do so. Some of the questions for the practitioners were constructed with inspiration from similar studies [24].

It was estimated that both questionnaires would take 5-10 minutes to complete.

Data from both questionnaires were treated anonymously, and the participants were made aware of consenting to the use of data by answering the questionnaires.

### The Citizens

**Figure 1:**
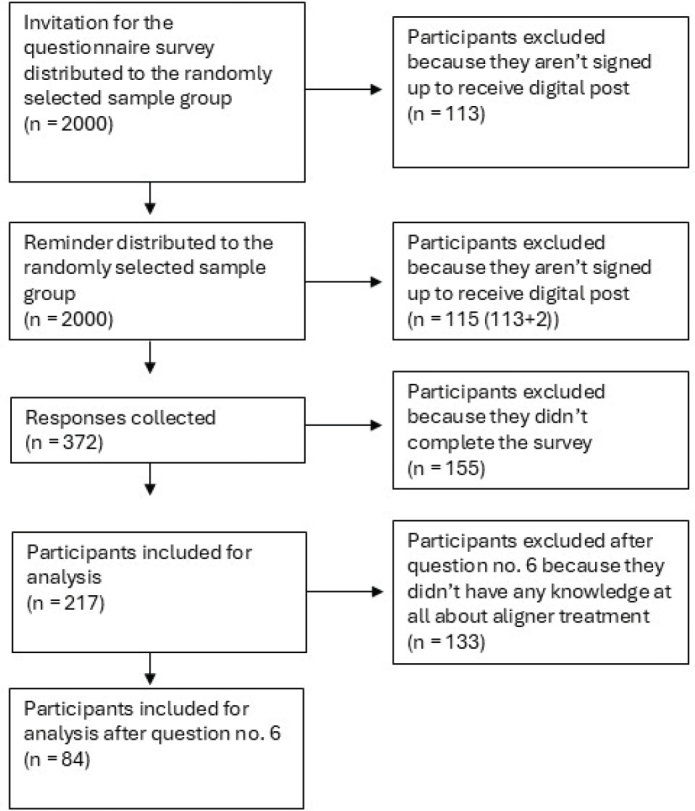
Distribution of the questionnaire for the citizens.

The group of citizens was chosen by randomised sampling through the Danish Health Data Authority. A calculation of strength was performed in cooperation with Statistics Denmark and was set to 2000 individuals. Full names and civil registration numbers of the 2000 citizens were requested in order to distribute the invitation to the questionnaire through Digital Post (Figure 1).

### The Practitioners

**Figure 2:**
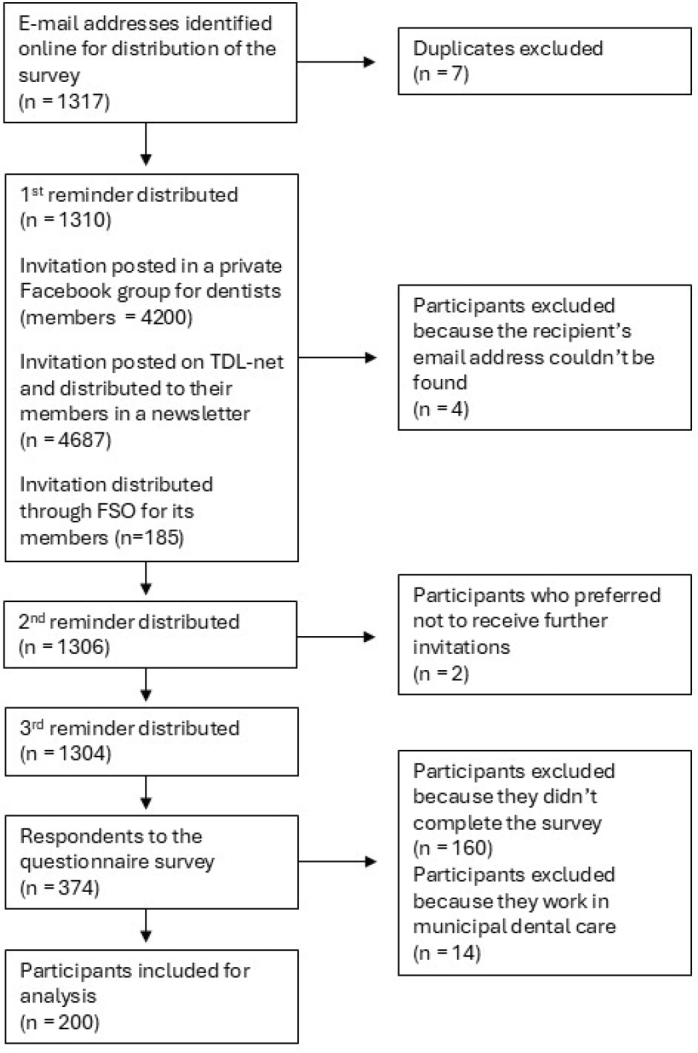
Distribution of the questionnaire for the practitioners.

The group of practitioners was chosen by purposive sampling method where the questionnaire was distributed to 1317 dental clinics whose e-mail addresses were available online. In case the e-mail address was not available online, it was sought retrieved by telephone (Figure 2).

### Statistical Considerations

Multiple data-driven groupings were performed due to the number of respondents included for analysis (Tables 1 and 2).

**Table 1:** Grouping of data, practitioners.

Question	Grouping of data
Question 4	‘Years working in practice’ was grouped in intervals of 10 years.
After question 5	Responses from respondents belonging to ‘Oral and maxillofacial surgeon’ or ‘Other education’ were excluded for the rest of the analysis because they did not meet the inclusion criteria.
Question 6–10	These questions were grouped with the extremes separate and otherwise in intervals of 25%.
Question 12–15+17	The participants were able to answer according to a 5-step Likert scale which was grouped down to 3 steps because of the low number of respondents in the groups. ‘Always’ and ‘Often’ were combined and ‘Rarely’ and ‘Never’ were combined.
Question 18	The answers were combined as ‘100%’ to show how many of the participants who always would edit in the digital treatment plan and thereafter as ’50–99%’, ‘1–49%’ and ‘0%’.
Question 24	Regarding how often retention is used after CAT, the answers varied more than in question 18 why they were grouped as ‘100%’ to show how many of the participants who always use retention after CAT and thereafter as ’70–99%’, ’30–69%’ and ‘0–29%’.

**Table 2:** Grouping of data, citizens.

Question	Grouping of data
Question 1	Age was grouped even further into three age groups: 18–30 years, 31–50 years and 50+ years.
Question 4	Regarding level of education ‘Vocational education’, ‘Higher general examination’ and ‘Short-cycle higher education’ were combined to ‘Short education’
Question 5	Regarding income ‘< 150.000’ and ‘150.000–349.999’ were combined to ‘Low income’. ‘350.000–549.999’ and ‘550.000–749.999’ were combined to ‘Average income’. ‘750.000–949.999’ and ‘> 950.000’ were combined to ‘High income’.
Question 6	Data from the five categories were grouped into four categories where ‘To a very high degree’ and ‘To a high degree’ were combined. This question could not be grouped to a 3-step Likert scale because it was of importance if the citizens answered ‘Not at all’ regarding their knowledge to CAT why this was kept as a separate category.
Question 7, 8, 10a–13a, 19a, 21a, 11b–15b and 11c–14c	The participants were able to answer according to a 5-step Likert scale which was grouped down to 3 steps because of the low number of respondents in the groups. ‘Always’ and ‘Often’ were combined and ‘Rarely’ and ‘Never’ were combined.

Data were treated by descriptive statistics using IBM SPSS Statistics (v. 29.0 for Windows). Due to a significant difference in answers between the practitioners, a chi-square test was performed regarding the use of a lateral cephalogram. The level of significance was set to p<0.05.

## Results

### The Practitioners

In the practitioner group, 374 individuals responded, of which 160 were excluded because the questionnaire was not completed. A further 14 respondents were excluded because they clearly stated working in municipal dental care, which left 200 respondents for inclusion in the study (Figure 2). Background information on the practitioners is shown in Table 3. Mean time working in practice was 24.7 years (± 12.4 years). Twenty-three percent (n = 39) of the general dentists answered ‘Yes’ when asked about offering CAT while 57% (n = 13) of the orthodontists offered CAT (Table 4).

**Table 3:** Background information, practitioners.

	% (n)
Sex	
Female	68 (136)
Male	32 (64)
Other/prefers not to answer	0 (0)
Age (years)	
20–30	5 (9)
31–40	15 (29)
41–50	31 (62)
51–60	21 (42)
61–70	24 (48)
70+	5 (10)
Educational background	
General dentist	87 (173)
Maxillofacial surgeon	1 (1)
Orthodontist	12 (23)
Other	2 (3)
**Total**	100 (200)

**Table 4:** Is CAT a treatment that you offer to your patients and perform yourself?

	Educational background				
	General dentist	Maxillofacial surgeon	Orthodontist	Other	Total
	% (n)	% (n)	% (n)	% (n)	% (n)
Yes	23 (39)	0 (0)	57 (13)	67 (2)	27 (54)
No	77 (134)	100 (1)	43 (10)	33 (1)	73 (146)
**Total**	100 (173)	100 (1)	100 (23)	100 (3)	100 (200)

#### Practitioners offering CAT

Table 5 shows how often the practitioners’ patients have an indication for CAT, how often the patients demand CAT, how often the patients are advised not to receive CAT or how often the patients reject CAT even though there’s an indication for the treatment.

**Table 5:** Practitioners offering CAT.

		Educational background		
		General dentist	Orthodontist	Total
		% (n)	% (n)	% (n)
How many of your patients have indication for CAT during an average year?	None - < 1%	0 (0)	8 (1)	2 (1)
	1–10%	46 (18)	38 (5)	44 (23)
	11–25%	41 (16)	38 (5)	40 (21)
	26–50%	5 (2)	15 (2)	8 (4)
	51–75%	5 (2)	0 (0)	4 (2)
	> 75%	3 (1)	0 (0)	2 (1)
Among all your patients, how many demand CAT on their own initiative?	None - < 1%	13 (5)	0 (0)	10 (5)
	1–10%	62 (24)	23 (3)	52 (27)
	11–25%	15 (6)	8 (1)	13 (7)
	26–50%	3 (1)	54 (7)	15 (8)
	51–75%	3 (1)	8 (1)	4 (2)
	> 75%	5 (2)	8 (1)	6 (3)
How often do you advise patients who demand CAT not to go through treatment?	None - < 1%	5 (2)	0 (0)	4 (2)
	1–10%	67 (2)	8 (1)	52 (27)
	11–25%	8 (3)	23 (3)	12 (6)
	26–50%	15 (6)	38 (5)	21 (11)
	51–75%	3 (1)	23 (3)	8 (4)
	> 75%	3 (1)	8 (1)	4 (2)
How many of your patients with indication for CAT choose not to go through with the treatment?	None - < 1%	21 (8)	8 (1)	17 (9)
	1–10%	28 (11)	46 (6)	33 (17)
	11–25%	21 (8)	31 (4)	23 (12)
	26–50%	23 (9)	15 (2)	21 (11)
	51–75%	5 (2)	0 (0)	4 (2)
	> 75%	3 (1)	0 (0)	2 (1)
**Total**		100 (39)	100 (13)	100 (52)

When asked about indications for CAT, both groups of practitioners agree that about half of all CATs are based on cosmetic indications. However, the practitioners disagreed about functional indication, which the general dentists more often treated with CAT (general dentists: mean 22.3% of cases; orthodontists: mean 7.2% of cases).

In general, the practitioners agreed on what material they collect prior to CAT except for a lateral cephalogram, which is more often included by the orthodontists (85%, n = 11 ‘always/often’) compared to the general dentists (5%, n = 2 ‘always/often’). This difference was statistically significant (p < 0.001).

In the orthodontist group, 100% of the respondents answered that an orthodontist is responsible for diagnostics and treatment planning, while this was not always the answer given by general dentists; hence, sometimes orthodontists were not involved in diagnosis and treatment planning.

The orthodontists most often replied that they always edit the digital treatment plan (62%, n = 8) and often make greater changes to the digital treatment plan compared to the general dentists (28%, n = 11). The majority of the general dentists make changes to the digital treatment plan in 50-99% of the cases.

#### Practitioners not offering CAT

The general dentists who do not provide CAT most often answered that it was due to “lack of experience or education” while the orthodontists not providing CAT most often were of the opinion that “fixed appliance achieves better treatment results”. By the practitioners who elaborated their response in free text, it was often noted that they believed CAT ought to be performed by specialists exclusively (31%, n = 45).

### The Citizens

Of the 2000 citizens to whom an invitation for the questionnaire was addressed, 372 responded. Due to non-completion of the questionnaire, 155 respondents were excluded, which left 217 for inclusion in the analysis (response rate = 11%). However, a further 133 respondents had to be excluded after question 6 because they indicated not to have any awareness of CAT why it was deemed irrelevant to include these citizens’ opinions on CAT. Therefore, after question 6, there were 84 respondents included for analysis.

In Table 6, demographics of the included respondents are shown.

**Table 6:** Citizen demographics.

	% (n)
Age (years)	
18–30	13 (28)
31–50	24 (51)
50+	64 (138)
Sex	
Female	56 (122)
Male	43 (94)
Other/prefers not to answer	0.5 (1)
Province	
Sealand and Bornholm	44 (95)
Funen	9 (19)
Jutland	47 (103)
Faroe Islands and Greenland	0 (0)
Educational background	
Basic school	8 (17)
Short-cycle higher education	31 (68)
Medium-cycle higher education	32 (69)
Long-cycle higher education	28 (61)
Other	1 (2)
Income (gross, DKK)	
Low	44 (95)
Average	44 (96)
High	0 (0)
Prefers not to answer	12 (26)
**Total**	100 (217)

#### Awareness of CAT among the Citizens

The respondents in the age group 18-30 years were the ones most aware of CAT, and the age group 50+ were least aware of CAT. The 18–30-year-olds most often became aware of CAT through their own dentist, and in case they wanted more information about CAT, they also got it through their own dentist. The 31–50-year-olds most often became aware of CAT through friends/family or their own dentist while the 50+ group most often gained their knowledge through friends/family only.

When asked about receiving CAT, 21% (n = 18) answered ‘Yes’, and the majority of these belonged in the 18-30 years group. Of those who had not received CAT, 24% (n = 16) stated to be ‘Undecided’, while 76% (n = 50) stated not to have any interest in CAT whatsoever.

#### Citizens who received/are receiving CAT

The citizens who chose CAT were most often influenced by themselves (76%, n = 13 ‘to a high degree’), and the reason for choosing CAT was mostly due to aesthetics (82%, n = 14 ‘to a high degree’). Aesthetics were also most often the reason why the practitioner had recommended CAT according to the citizens (71%, n = 12 ‘to a high degree’). Almost half of the respondents (47%, n = 8) replied that they would have wanted to receive orthodontic treatment even if CAT had not been a treatment option.

#### Citizens who are undecided regarding CAT

Regarding the reason why these citizens were undecided about CAT, the 18–30-years-olds (100%, n = 3 ‘agree’) and the 31–50-years-olds (73%, n = 8 ‘agree’) stated that this was due to economics while the reasons in the 50+ group varied more. It was rarely due to concerns about health risks/side effects (31%, n = 5 ‘agree’), considerations about alternative treatment options (19%, n = 3 ‘agree’) or because their practitioner advised against CAT (31%, n = 5 ‘agree’) that this group of citizens were undecided about CAT. Aesthetics was most often the reason why these citizens had considered CAT (75%, n = 12 ‘to a high degree’) and why their dentist had recommended the treatment (38%, n = 6 ‘to a high degree’).

#### Citizens who have no Interest in CAT

In general, this group of citizens had not at any time felt a need or desire for CAT (92%, n = 46 ‘to a low degree/not at all’).

### Mutual Questions for Practitioners and Citizens

Table 7 shows how well informed the citizens felt regarding certain parameters. The practitioners in general agreed to provide sufficient information to their patients prior to CAT.

**Table 7:** Information prior to CAT, citizens.

		Age group		
		18–30	31–50	Total
		% (n)	% (n)	% (n)
Purpose of the treatment	To a high degree	55 (6)	67 (4)	59 (10)
	In some degree	36 (4)	17 (1)	29 (5)
	To a low degree/not at all	9 (1)	17 (1)	12 (2)
Practicalities	To a high degree	55 (6)	83 (5)	65 (11)
	In some degree	36 (4)	0 (0)	24 (4)
	To a low degree/not at all	9 (1)	17 (1)	12 (2)
Treatment duration	To a high degree	64 (7)	83 (5)	71 (12)
	In some degree	27 (3)	0 (0)	18 (3)
	To a low degree/not at all	9 (1)	17 (1)	12 (2)
Retention	To a high degree	36 (4)	33 (2)	35 (6)
	In some degree	45 (5)	50 (3)	47 (8)
	To a low degree/not at all	18 (2)	17 (1)	18 (3)
Oral hygiene	To a high degree	55 (6)	83 (5)	65 (11)
	In some degree	36 (4)	0 (0)	24 (4)
	To a low degree/not at all	9 (1)	17 (1)	12 (2)
Side effects and risks	To a high degree	18 (2)	17 (1)	18 (3)
	In some degree	18 (2)	67 (4)	35 (6)
	To a low degree/not at all	64 (7)	17 (1)	47 (8)
Consequences if no treatment	To a high degree	27 (3)	17 (1)	24 (4)
	In some degree	27 (3)	67 (4)	41 (7)
	To a low degree/not at all	45 (5)	17 (1)	35 (6)
Alternative treatment options	To a high degree	9 (1)	17 (1)	12 (2)
	In some degree	9 (1)	50 (3)	24 (4)
	To a low degree/not at all	82 (9)	33 (2)	65 (11)
Cost of the treatment	To a high degree	45 (5)	83 (5)	59 (10)
	In some degree	0 (0)	0 (0)	0 (0)
	To a low degree/not at all	55 (6)	17 (1)	41 (7)
Pictures with examples of before/after CAT from other treatments	To a high degree	18 (2)	50 (3)	29 (5)
	In some degree	27 (3)	17 (1)	24 (4)
	To a low degree/not at all	55 (6)	33 (2)	47 (8)
Visualization of the expected result	To a high degree	45 (5)	67 (4)	53 (9)
	In some degree	0 (0)	0 (0)	0 (0)
	To a low degree/not at all	55 (6)	33 (2)	47 (8)
Other	To a high degree	0 (0)	0 (0)	0 (0)
	In some degree	0 (0)	0 (0)	0 (0)
	To a low degree/not at all	100 (11)	100 (6)	100 (17)
**Total**	100 (11)	100 (6)	100 (17)	

The general dentists were often more satisfied with CAT (on average ‘highly satisfied’ in 78% of cases) compared to the orthodontists (on average ‘highly satisfied’ in 51% of cases). In part, this is in correspondence with the statement from the citizens that their dentist had been satisfied with the treatment result (76%, n = 13 ‘satisfied’). The practitioners agreed that most patients are ‘highly satisfied’ with their treatment result after CAT (general dentists: 89% of patients; orthodontists: 72% of patients), which the citizens also indicate to be (82%, n = 14 ‘satisfied’).

The practitioners found that ‘the achieved result is not consistent with the expected result’ (44%, n = 23), ‘the patient had poor compliance’ (38%, n = 20), ‘the used number of aligners exceeded the expected number of aligners’ (40%, n = 21) and ‘finish was difficult to achieve with aligners’ (35%, n = 18) as the main challenges with CAT. The orthodontists also found a challenge in ‘need for multiple adjustments/refinements in the treatment plan during treatment’ (38%, n = 5). When asked about what the practitioners found to be challenging in CAT for the patients, ‘the achieved result is not consistent with the expected result’ (31%, n = 16), ‘treatment duration’ (33%, n = 17), ‘the used number of aligners exceeded the expected number of aligners and because of that the treatment was prolonged’ (27%, n = 14) and ‘poor compliance’ (23%, n = 12) were among the most frequent reasons. According to the citizens, ‘discomfort or pain related to CAT’ was the most frequent reason for dissatisfaction with CAT (18%, n = 3). However, most of the citizens chose ‘irrelevant, I’m completely satisfied with the treatment and treatment result’ (29%, n = 5) when asked about dissatisfaction with CAT.

## Discussion

Recently, the interest in CAT has been increasing [1]. To the best of our knowledge, only few studies investigating the awareness and assessment of CAT have been performed. No studies investigating the awareness among the citizens in Denmark or how they experience the treatment have been performed. Neither has it been investigated how the general dentists and orthodontists assess CAT. Therefore, it was found relevant to study the awareness of CAT among the citizens as well as how the practitioners assess CAT.

### The Citizens

In this study, the age group most aware of CAT was the 18–30-year-olds. This is in accordance with previous findings in the literature where the patients demanding and receiving CAT often belong to the younger generations [4, 9, 25, 26]. Three studies [4, 9, 26] find that the reason for patients seeking CAT most often is due to aesthetics. This is in agreement with the present study where 83% of the respondents who had received/are receiving CAT indicated that aesthetic concerns played a major role in their decision about CAT. In contemporary society, appearance is of great importance with social media exposing us to constant self-presentation and comparison with others [27]. Many people might feel that they must change their physical appearance to “live up to” western beauty standards which we are being exposed to at an immense extent through various media platforms [27]. This, along with the growing social acceptance of cosmetic treatment and surgery - especially among younger generations - and the increasing number of influencers sharing their personal experiences online, is most likely pushing the demand for aesthetic treatment [27]. The results in the present study confirm the hypothesis that the need for CAT most often is based on cosmetics/aesthetics.

In the present study, the citizens had often gained awareness and knowledge about CAT through their friends/family or through their own dentist. This was partly in disagreement with the expectations and the hypothesis that patients often gain their knowledge about CAT through other sources than healthcare professionals. Contrary to this study where media and advertising seemingly were not the most influential sources other studies [26, 28–32] find that these sources are extremely influential when it comes to the citizens’ need for CAT. However, a single study [33] confirmed that dentists as well as patients’ social circles can play a major role in influencing the growing demand for CAT. The disagreement in previous and present results may be due to that an individual’s needs can be influenced by both media and healthcare professionals [23], and perhaps the citizens included in this study truly were less influenced by media and advertisements. However, there is a slight possibility that the answers regarding this subject might be influenced by “social desirability bias” [34] where the included citizens might have preferred to appear less influenced by media and advertisement than what they really are. This study is, however, not designed to genuinely point out how influential the media is regarding CAT in Denmark.

In the present study, the citizens who had received or were undergoing CAT had often made this decision based on their own opinion. This is in accordance with several studies [4–10, 26, 33, 35] that emphasise the importance of aesthetics in CAT. Contrary to the findings in the above-mentioned studies [4–10, 26, 33, 35] where CAT often was preferred to FAT by the patients due to aesthetics, the citizens in the present study indicated that they would have wanted to receive orthodontic treatment even though CAT had not been an option. This might indicate that for most patients the final treatment result is of great importance and that they are willing to compromise on aesthetics and possible inconveniences during an orthodontic treatment.

The findings about where the citizens get their knowledge about CAT and why they were interested in CAT support the idea that need, demand and supply are influenced by several factors such as family, friends and health professionals [23].

Regarding information prior to CAT, there was in general agreement between what the citizens felt they had been informed about and what the practitioners stated to inform about. Despite this, a discrepancy was noted between the citizens and the practitioners regarding alternative treatment options, possible side effects and health risks and consequences if no treatment is carried out. Whether this is because the practitioners put less emphasis on these subjects when informing about CAT or whether the citizens tend not to remember this kind of information (recall bias [36]) cannot be confirmed in this type of study. A previous study [37] found that online information about CAT is of very poor quality, which may make the decision about CAT difficult. A patient’s consent to treatment cannot be seen as valid in case they have not been sufficiently informed prior to treatment [37]. In particular, the citizens felt very poorly informed about alternative treatment options. According to the Danish Health Care Act [19], the patients should have been informed about this in order to decide whether to receive treatment or not on a sufficiently informed basis.

In accordance with the existing literature [12, 38, 39], the citizens in this study were in general satisfied with their CAT. A single younger citizen had been dissatisfied with the treatment allegedly due to permanent physical damage. Unfortunately, it was not possible to follow up on what sort of damage the citizen had experienced due to the design of the study.

Regarding the practitioners, the degree of satisfaction with CAT was a bit lower compared to the citizens. The general dentists were mostly more satisfied with CAT compared to the orthodontists. This might be due to the expertise among orthodontists with orthodontic treatment and perhaps a different standard for what they perceive as a successful treatment result. Another explanation might be that the orthodontists use CAT to treat more complex cases compared to the general dentists. Another study [12] also finds a discrepancy in the level of satisfaction between the patients and practitioners and that orthodontists are more critical about the treatment results from CAT [40].

### The Practitioners

#### Who is performing CAT?

Around half of the orthodontists were offering CAT while only 23% of the general dentists were offering the treatment which is low compared to another study [41]. However, this distribution is to be expected given that the orthodontists are specialists in orthodontics.

In previous studies [41, 42], it has been shown that general dentists do not feel very well educated in performing orthodontic treatments after graduating their dental education. To avoid unintentional accidents during treatment, being supervised by a specialist might help [14]. Furthermore, the more experienced and educated the practitioner is, the better he/she is at assessing the complexity of the dental movements needed in the moderate to complex cases [41]. One study [14] finds that the more clinical experience a practitioner has with CAT. the more is he/she likely to obtain the planned movements and with better prediction. In the present study, the orthodontists were not as prone to perform CAT solely based on a functional indication compared to the general dentists, which might indicate a difference in their ability to assess the complexity of a case. Another study [43] showed that the general dentists were more prone to perform CAT in complex cases; the authors noted that the general dentists were more likely to use CAT for treating severe crowding without performing extractions compared to the orthodontists in the study. This could lead to a profuse proclination of the teeth and expansion of the arch, which would give an unstable result and in worst case expand the teeth out of the alveolar bone. The authors [43] noted that the results from their study might indicate that general dentists could be more focused on an aesthetically pleasing result while the orthodontists are more focused on the occlusion and function alongside the aesthetics.

In cases where the only reason for CAT is achieving better smile aesthetics, the practitioner must assure that the patient is sufficiently informed about potential health risks and side effects related to the treatment. According to Doyal and Gough [44], humans are not always aware that the needs we have might be harmful to us why it is indeed important that the practitioner makes sure that the patient is aware of and accepts potential side effects. Besides, the dental practitioners must make up their mind whether they find that they are up for the task. CAT does not differ from any other kind of orthodontic treatment where thorough diagnostics and treatment planning are essential in avoiding mistreatment.

#### Lateral Cephalograms

A significant difference in the inclusion of a lateral cephalogram as part of the diagnostic material prior to CAT between the two groups of practitioners was found. It has been indicated [18] that a lateral cephalogram ought to be included prior to orthodontic treatment and especially if the practitioner is lacking experience in orthodontics. Another study [45] shows that orthodontists include this radiographic material in their diagnostics in most cases. The treatment plan might even alter based on the inclusion of a lateral cephalogram [46]. The complexity of a malocclusion can indeed be very difficult to determine without a lateral cephalogram due to determining whether a malocclusion is of dentoalveolar or skeletal origin. Without this assessment, there may be a risk of incorrect treatment, which in worst case could place the patient in a worse situation compared to their starting point. Some malocclusions of dentoalveolar origin can be eliminated using CAT while the same type of malocclusion of skeletal origin cannot, which stresses the need to include the right radiographic material prior to CAT [43].

#### Digital Treatment Planning

In the present study, the orthodontists edited more and made more comprehensive corrections in the digital treatment plan compared to the general dentists. This might be because orthodontists more often treat more complex cases with CAT than the general dentists. Another cause might be that the general dentists simply lack experience in orthodontic treatments and therefore may not recognise the complexity of a case and the need for editing the digital treatment plan. This along with the differences in collection of diagnostic material prior to CAT might point towards a confirmation of the hypothesis that not all general dentists have sufficient professional knowledge and competences about CAT. In a recent study [47], it was noted that the orthodontists in most cases made up to 14 changes to the initial digital treatment plan prior to accepting it. According to the authors, that shows just how much expertise it takes to reach an acceptable treatment plan for each patient. Another study [43] also found that the orthodontists spend more time assessing and editing each patient’s digital treatment plan compared to the general dentists.

The software used for digital treatment planning in CAT is meant to visualise the treatment for the practitioner, but it will always be the practitioner’s responsibility to validate this prior to treatment [18, 47].

#### Responsibility for Diagnostics and Treatment

Regarding responsibility for CAT, the general dentists answered that often it would be themselves as non-specialists followed by ‘a combination of the dentist and clear aligner provider’. In CAT, the treating dentist is the only one held responsible for the treatment, diagnostics and treatment planning [3, 18]. Concerning the educational level of the practitioner responsible for the treatment, most of the general dentists replied that it was a general dentist with specific training in CAT. However, a few of the general dentists in the present study replied that they did not have any specific training or education regarding CAT but nevertheless offered the treatment to their patients. Without sufficient education and knowledge, there may be a risk of mistreating the patients due to a lack of ability to assess the complexity of a case and thereby make the right decisions in planning a treatment [14, 41].

#### Practitioners who do not provide CAT

In the present study, there were both orthodontists and general dentists who did not provide CAT. Among the orthodontists, it was often due to the opinion that FAT achieves better results, which is in accordance with another study [24]. According to several studies [1, 16, 20, 48, 49], CAT is still to be considered inferior to FAT in most movements when it comes to predictability and effectiveness.

In the present study, most of the general dentists did not provide CAT due to lack of experience or education, and they believed that CAT should be performed by specialists. This is in agreement with previous studies [41, 42] that noted that general dentists did not feel sufficiently educated regarding orthodontics when graduating dental school. A 2010 survey [50] even demonstrated that most orthodontists and general dentists did not feel confident in CAT and in understanding how it works after their initial certification by Invisalign®.

### Limitations and Bias

A limitation of the present study is the low response rate, especially in the citizen group where only 84 respondents were included out of the 2000 citizens who received an invitation for participation. This introduces the risk of non-response- and selection bias [51, 52] and critically limits the population-level generalisability. However, the number of respondents included for analysis in the practitioner group (n = 200) was comparable to similar studies [11, 14, 50]. Due to the number of respondents, only a single statistical test was performed since the strength of such tests often was deemed too low and the risk of it being misleading too high. Dropout analysis was not performed.

Another limitation of the study is that all data in the study is based on self-reported answers. This should be considered in relation to the findings and conclusions of the study. Furthermore, the questionnaires were only available in Danish, which limited the accessibility to participate in the survey.

A strength in the present study was the ability to reach a large group of practitioners and citizens [53]. Both questionnaires went through pilot testing prior to distribution. Feedback was evaluated and the questionnaires revised, which can be observed as a way of preventing misunderstandings among the respondents.

In the citizen group, a population was randomly chosen by an outside organisation (the Danish Health Data Authority) why the risk of selection bias is to be considered low. Yet, in the practitioner group, dentists cannot be compared to dentists who did not wish to participate. Whether representation is achieved is difficult to assess. Perhaps practitioners with a significant interest in CAT as well as those with a very negative attitude towards it are more likely to participate than those with no interest in the subject or those who are more uncertain about their attitude towards it.

## Conclusion

This study has provided valuable knowledge on the awareness and satisfaction of CAT among the citizens and indicated how practitioners assess CAT of adult patients in private dental practice in Denmark.

Conflicting information about whether patients are sufficiently informed or not prior to CAT and indications that the different groups of practitioners assess diagnostics and treatment planning in CAT differently could potentially point out an issue and should be closer investigated.

The results presented in this study could be the foundation for hypotheses in future studies in the field.

## Data Availability

The data underlying this article will be shared upon reasonable request to the corresponding author.
